# Bone remodeling effect of a chitosan and calcium phosphate-based composite

**DOI:** 10.1093/rb/rbz009

**Published:** 2019-03-18

**Authors:** Lilja Kjalarsdóttir, Arna Dýrfjörd, Atli Dagbjartsson, Elín H Laxdal, Gissur Örlygsson, Jóhannes Gíslason, Jón M Einarsson, Chuen-How Ng, Halldór Jónsson

**Affiliations:** 1 Department of Orthopaedic Surgery, Landspítali University Hospital, Reykjavík, Iceland; 2 Faculty of Medicine, University of Iceland, Reykjavík, Iceland; 3 Genís hf., Siglufjördur, Iceland; 4 Department of Materials, Biotechnology and Energy, Innovation Center Iceland, Reykjavík, Iceland; 5 Department of Vascular Surgery, Landspítali University Hospital, Reykjavík, Iceland

**Keywords:** bone remodeling, bone implant, bone defects, chitosan, degree of deacetylation, micro-CT, rat mandible

## Abstract

Chitosan is a biocompatible polymer that has been widely studied for tissue engineering purposes. The aim of this research was to assess bone regenerative properties of an injectable chitosan and calcium phosphate-based composite and identify optimal degree of deacetylation (%DDA) of the chitosan polymer. Drill holes were generated on the left side of a mandible in Sprague-Dawley rats, and the hole was either left empty or filled with the implant. The animals were sacrificed at several time points after surgery (7–22 days) and bone was investigated using micro-CT and histology. No significant new bone formation was observed in the implants themselves at any time points. However, substantial new bone formation was observed in the rat mandible further away from the drill hole. Morphological changes indicating bone formation were found in specimens explanted on Day 7 in animals that received implant. Similar bone formation pattern was seen in control animals with an empty drill hole at later time points but not to the same extent. A second experiment was performed to examine if the %DDA of the chitosan polymer influenced the bone remodeling response. The results suggest that chitosan polymers with %DDA between 50 and 70% enhance the natural bone remodeling mechanism.

## Introduction

Successful healing of bone tissue defects depends on several factors. In clinical situations, where spontaneous healing is not achieved by internal factors alone, external factors must be applied to stimulate the healing process [1]. Healing in such cases is stimulated by implants (IPs) providing osteogenic, osteoconductive and/or osteoinductive properties, where bone grafts such as autografts, allografts or synthetic graft substitutes are commonly used [2]. Autografts are currently regarded as the golden standard in orthopedic surgery. However, donor site morbidity is a serious drawback of the procedure [3]. Synthetic grafts containing recombinant growth factors such as bone morphogenic protein have been shown to be effective in many applications [4]. However, because of safety concerns they are only approved for a limited number of indications [5]. Hence, an intensive search for replacement therapies is ongoing, involving development of various types of innovative graft substitutes acting as scaffolds that guide and promote new bone growth [6].

Chitosan is a natural, biodegradable and biocompatible polymer that has a potential to act as a scaffold that promotes bone healing [7]. Chitosan is a linear polysaccharide comprising randomly distributed β-(1 → 4)-linked D-glucosamine and *N*-acetyl-D-glucosamine units. Chitosan is produced by deacetylating chitin, an insoluble linear β-(1 → 4)-linked *N*-acetyl-D-glucosamine polysaccharide that serves as a structural material in the exoskeleton of arthropods and cell walls of fungi. The deacetylation process exposes positively charged amine groups of the glucosamine units which increases solubility of the polymer [8]. *In vitro* studies suggest that chitosan has osteogenic properties as it promotes differentiation of stem cells into bone-forming osteoblasts and promotes growth of bone colonies [9]. *In vivo* studies suggest that chitosan alone is sufficient to stimulate osteogenesis [10, 11]. In some previous studies, other materials with osteogenic, osteoinductive and/or osteoconductive properties were added to chitosan-based composites to further stimulate the bone healing process [12, 13]. The IPs used in the present study were a combination of a chitosan polymer and a blend of tetracalcium phosphate (Ca_4_(PO_4_)_2_O) and α-tricalcium phosphate (α-Ca_3_(PO_4_)_2_). Calcium phosphate-based bone cement was chosen as a base for the IPs because of its widespread clinical use and because it has been shown to be biocompatible as well as osteoconductive [14].

The aim of the study was to assess the osteogenic properties of chitosan and calcium phosphate-based IPs in a model of critical bone lesion in the rat mandible. In a first set of experiments, osteogenic properties were assessed as a function of time using a single type of chitosan polymer. In a second set of experiments, the optimal degree of acetylation of the chitosan polymer was determined.

## Materials and methods

### Implants

Kits of the IP formulation were produced at the laboratories of Genis hf., Iceland, consisting of 3.0% w/v of chitosan polymers with different degree of deacetylation (96% DDA, 70% DDA and 50% DDA) (Genis hf., Iceland), 30.9% w/v tetracalcium phosphate/α-tricalcium phosphate (Himed, USA), 4.2% w/v sodium glycerophosphate (Merck, Germany), 1.9% w/v calcium hydroxide (Sigma-Aldrich, USA), 7.7% w/v phosphoric acid (Merck) and water. The kits were sterilized by 20 kGray γ-irradiation at Radiation Center, Oregon State University, Corvallis, OR, USA. Directly after mixing solid and liquid components, the IP material is soft and injectable, but sets into a non-loadbearing solid within 24 h, with increasing hydroxyapatite content in the further course of the setting reaction.

### Animals and experimental design

First, time-dependent series: twenty-eight healthy Sprague-Dawley rats (Tactonic, Denmark) were assigned to one of the following two treatment groups: sham control (*n* = 12/group) and 70% DDA chitosan composite IP (*n* = 16/group). These groups were then divided into four subgroups of three animals for sham control subgroups and four animals for chitosan composite IP subgroups with different durations of life phase: 7, 10, 14 and 22 days. One animal from the 22 day group died in surgery and was used as an example for Day 0.

Second series testing the effect of %DDA of the chitosan polymer in the IP: 36 healthy Sprague-Dawley rats (Tactonic, Denmark) were assigned to one of the following four treatment groups: sham control (*n* = 10/group); 50% DDA chitosan composite IP (*n* = 12/group); 70% DDA chitosan composite IP (*n* = 6/group) and 96% DDA chitosan composite IP (*n* = 8/group). All animals were sacrificed 7 days post-operatively. The observation time of 7 days was chosen as the results from the time-dependent study had indicated highly dynamic growth at this time point and thus a high sensitivity of the model toward %DDA was expected.

Animal feed was obtained from Special Diet Services, Essex, England.

All experiments were designed and conducted according to the FELASA guidelines and approved by the Icelandic Veterinarian Scientific Ethical Authorities.

### Surgical procedure

**Figure 1:**
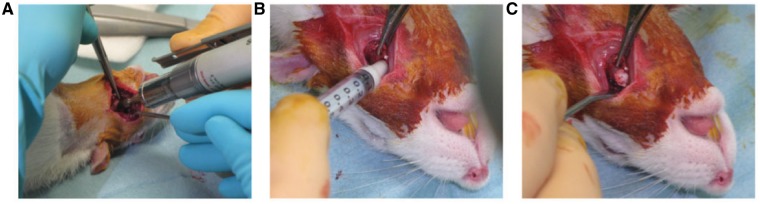
Overview of surgery in a rat receiving implant. (**A**) Dental drill was used to drill the hole. (**B**) Implant injected into the hole. (**C**) Appearance of tissue after implantation showing how implant fills the hole

The animals were anesthetized with Hypnorm/Dormicum and received prophylactic antibiotic treatment with trimethoprim 80 mg/ml, sulphadiazine 400 mg/ml, 0.0005 ml/g rat subcutaneous (s.c.) prior to surgery and 1 day post-operatively. To compensate for blood and fluid loss 5 ml of Ringers Acetate were injected subcutaneously. Skin of the jaw was disinfected with alcoholic iodine prior to surgery. The skin was incised longitudinally along the posterior edge of the mandible. The masseteric muscle was released subperiosteally from the edge of the bone and masseteric fossa exposed. A hole was drilled into the central part of the masseteric fossa with a 4-mm dental drill (Fig. 1A). Thereafter, bone debris was thoroughly rinsed from the drilled area by flushing with 5 ml sterile saline water. In the first experiment, the void was filled with 40 µl of the IP formulation using a syringe without an attached needle (Fig. 1B and C) or left untreated (control). In the second experiment the IP volume was decreased to 25 µl because 40 µl proved to be excessive as IP material tended to smear out around the drillhole. The surgical wound was closed with 4–0 polyglactin running s.c. suture and the skin with 4–0 polyamide nonabsorbable running suture. Blood loss was estimated by weighing the dental rolls used for soaking up blood during surgery. For post-operative analgesia, either Butorphanol 2 mg/kg or Buprenorphine 0.05 mg/kg, was given no later than 4 h after induction of anesthesia, or as soon as the animal started moving post-operatively. The time span for analgesic treatment was estimated on basis of behavior suggesting freedom from pain and distress. During surgery and until ambulatory, animals were kept on a heat mat under continuous observation, monitored for body temperature, consciousness, respiratory frequency as well as signs of pain and distress. Animals still showing distress symptoms after 48 h post-operatively, received further analgesic treatment as needed.

Surgery was well tolerated by all except one animal from the 22 days group, which died of excessive bleeding due to a rupture of the sublingual artery. One rat from the control group had a swollen neck for 3 days post-operation but body temperature was stable and within normal limits. All rats lost some weight during the first days after surgery but had reached their pre-operative body weight 1 week post-operatively. No other complications were recorded.

All animals were sacrificed by injection of an overdose of Hypnorm/Dormicum intraperitoneally. To secure death before dissection the aorta was cut off the heart. Left masseteric muscle was released from the mandible bone and inspected macroscopically for infection or necrosis. Thereafter the jaw bone was explanted and cleaned from soft tissue and the mandible placed in a fixation media containing 3.7% formaldehyde in a sodium phosphate buffer at pH 7. The most common macroscopic finding during autopsy was a pale discoloration of the inner surface of the masseteric muscle, in the area just above the drill hole in the mandibular bone, most probably caused by the dissection from the bone during the surgical procedure.

### X-ray micro-CT analysis

Scanning was performed in an x-ray micro computed tomography (CT)-scanner (Nanotom, General Electric Inspection Technologies). Samples were fixed in a closed plastic cylinder filled with 3.7% buffered formaldehyde solution and mounted on the rotational table in the CT-scanner. Scans were performed with an aluminum phantom and a plastic phantom (PET) as reference points for gray value comparison. Magnification was 4, voxel size 12.50 µm/voxel edge, number of images collected 1080 (step size 0.33°), with exposure time of 2000 ms, frame averaging of 3, and 1 frame skipped. X-ray settings were 100 kV, 80–85 and 125 µA, using tube mode 0 and no filter. Volume reconstruction was performed using the Datos-x software accompanying the CT-scanner. Data analysis was performed using Volume Graphics Studio Max 2.0 from Volume Graphics.

**Figure 2:**
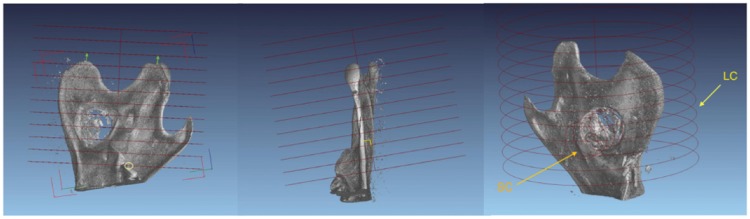
Standardization of a partial mandibular volume from micro-CT data according to anatomical landmarks in order to enable comparison of total bone volume. Orientation of the larger cylinder (LC) embracing the entire posterior part of the mandible. One end of the cylinder was positioned so as to sit on the innermost molar and the orientation of the cylinder was adjusted so as to be perpendicular to an imagined plane sitting on HM and the end of the angular process. The smaller cylinder (SC) also indicated covered the drillhole encompassing all remaining bone splinter as well as the implant

New bone appears darker on the grayscale than old bone since it has a lower mineral content. Old bone could be segregated from new bone by grayscale separation but since the IP composite exhibited similar grayscale values as new bone, the IP volume had to be subtracted to get a measure of new bone formation. A large cylinder (LC) of 9 mm radius and 14 mm length was defined which comprised the entire bone extending back from the hindmost molar, including the head of mandible, angle of mandible, coronoid process and the hole with or without an IP (Fig. 2). In all cases, a smaller cylinder (SC) was defined around the hole, perpendicular to the mandible. This cylinder was used to isolate the IP as well as the remaining bone splinters left in the hole. By defining a grayscale value interval, the combined volume of old bone, new bone and IP material within LC and SC was determined manually. Total bone volume (TBV) expressed in cubic millimeters was established as the sum of voxel volume defined by the specified grayscale value interval within LC, subtracted by the voxel volume measured in SC. In this way, an estimate of the TBV (new and old bone) of all jaws was obtained and comparison between treatment groups made possible.

### Histology

Histological evaluation was used to confirm the characterization of bone structures seen in the CT images, as old versus new bone. Specimens from mandibles were decalcified in hydrochloric Decalc solution (Histolab product AB, Sweden), for 30 min to 1 h. Then tissue specimens were put into cassettes and tissue rinsed in running tap water for 30 min. Next step was the conventional method of tissue processing overnight in an automatic tissue processor (Tissue Tek VIP, Sakura) ending with paraffin embedding where the tissue is embedded in paraffin wax at 63°C. Paraffin blocks were then moved over to a cooling plate for hardening and after that to a microtome for sectioning (Leica RM2255, Fully Automated Rotary Microtome). Disposable blades were used (Accu-Edge Disposable Blades, Sakura) for cutting sections at 3 µm thickness. Sections were mounted on coated microscope slides (Star Frost 76×26 mm, Knittel Glas GmbH, Germany), dried in a 60°C oven for 60 min. Ten sections were taken from each paraffin block at 12 µm intervals each and mounted on 10 Star Frost slides marked with specimen number and Roman numerals from I to X. After drying, H&E staining was performed using Shandon instant Hematoxylin and Eosin solution (Sigma-Aldrich).

### Statistical analysis

For statistical analysis SigmaPlot 13 (Systat Software, Inc.) was used. ANOVA and Student’s *t*-test (two tailed) were used for comparison of groups. The level of significance was accepted at *P* < 0.05.

## Results

### Observation of bone remodeling in response to IP

**Figure 3:**
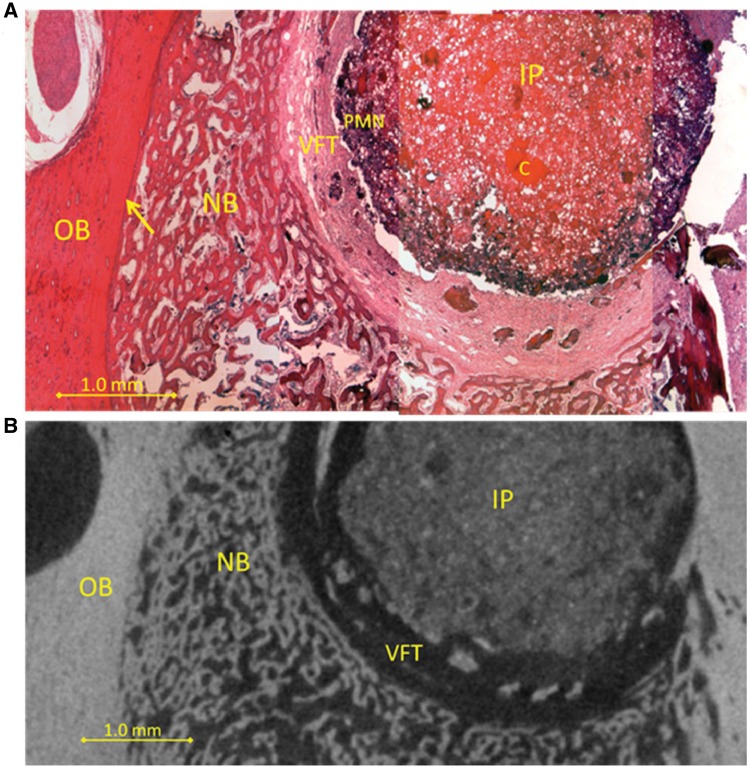
(**A**) Drilled hole with 14 days old implant (IP) containing PMN leukocytes (PMN) stained blue in the outer rim of the implant, then vascular fibrous tissue (FT), then new trabecular bone (NB). Original bone is on the left (OB) and the edge between old and new bone is indicated (arrow). Undissolved chitosan can be seen in the implant (C). (**B**) Virtual sectioning through a micro-CT image of the same sample. Drilled hole with implant (IP) then vascularized fibrous tissue (VFT, dark layer), then new trabecular bone (NB). Original bone (OB) is on the left

**Figure 4:**
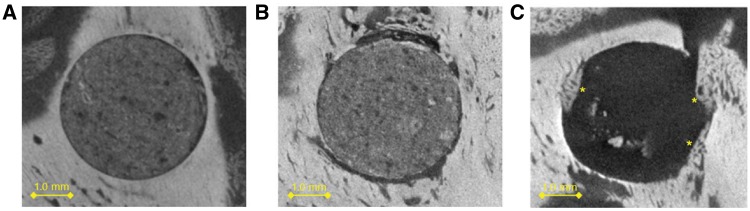
Micro-CT image sectioning of implant comparing Day 7 (**A**) and Day 22 (**B**). (**C**) Control sample at Day 22. No significant bone formation was observed in the implants. In the control sample, little new bone (*) formation was observed

Histological analysis showed polymorphonuclear (PMN) cells invading the outer edges of the IP which was surrounded by a vascular fibrous tissue (VFT). Outside the VFT, new bone tissue was observed, derived from bone remodeling of the old bone tissue (OB). Histological analysis of samples from all time points of the time-dependent experimental series revealed similar results; an example of how an IP looks 14 days after surgery is shown (Fig. 3A). By selecting micro-CT sections in the same plane as histological sections it was tested whether micro-CT analysis correlated with histological analysis. Using this approach it could be shown that these methods correlate well as both can distinguish between new and old bone (Fig. 3B). The appearance of the IP was similar throughout the time course examined and no significant new bone formation was observed in the IP at any of the time points (Fig. 4). Control animals with empty drill holes showed similar VFT formation surrounding the drill hole (histological data not shown), proximal bone remodeling and little new bone formation in the drill hole (Fig. 4C).

**Figure 5:**
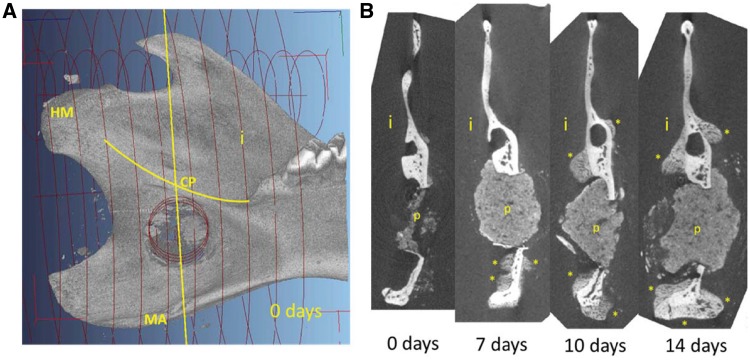
The effect of implant on mineralized tissue formation as a function of time in the rat mandible. (**A**, left): Overview of the interior side of the mandible indicating the drilled hole. Yellow line shows the location of the transverse section. (**B**, right): Four transverse sections of 0, 7, 10 and 14 days post operation showing the implant (p) and the formation of new mineralized tissue (*). i, interior side; MA, mandibular angle; CP, condylar process; MH, head of mandible. For the 0 days time point, rats were sacrificed at the same day as the operation

**Figure 6:**
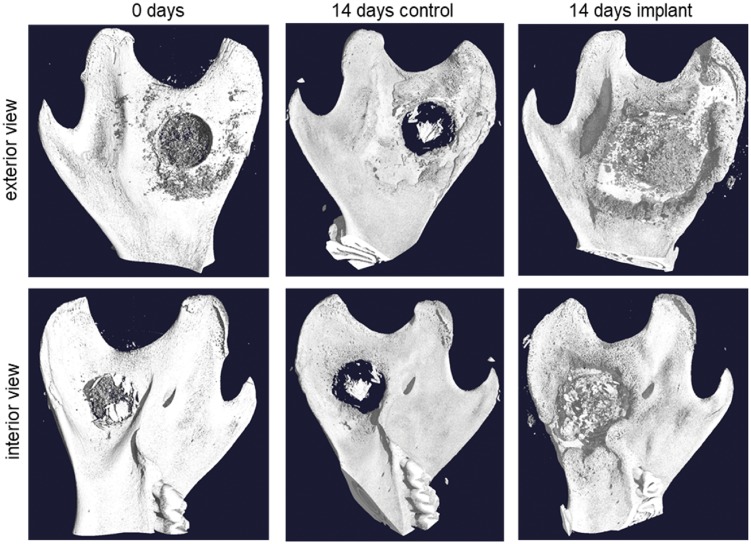
Three-dimensional reconstruction from micro-CT data comparing a control sample and a sample with implanted composite with 70% DDA chitosan after 14 days of incubation. The control sample (center) shows some thickening of the bone around the hole as a natural response to the injury. The sample with implant (right) shows a much more distinct thickening of the bone in the same areas around the hole. As a reference, a sample from an animal that died in surgery is shown (left). As the implant material did not have time to set, most of it was washed away, leaving some remnants around the hole

Since no new bone formation was observed at the site of the IP we extended our search and looked at the whole left side of the mandible using micro-CT. Comparative micro-CT analysis of this portion of the mandible suggested that new bone was forming along the mandibular angle (MA) and around the drill hole. Figure 5 shows transverse micro-CT sections of the mandible at several different time points and shows that new tissue has formed as early as 7 days after surgery. For clarity, the sample in Fig. 5B showing IP from Day 0 came from an animal that had only had the IP for a few hours, the IP had not settled and was therefore not fully preserved with the sample. For the other time points, the IP itself was proportionally large compared with the thickness of the bone and did not seem to undergo any degradation during the experimental period. On Day 7, partially mineralized new bone tissue was found in areas surrounding the hole and along the MA. On Days 10–22, volume of new bone had visibly increased around the hole, both on the interior and exterior sides of the mandible. Figure 6 shows a control sample and a sample with an IP 14 days after surgery. In response to the injury mineralized tissue is forming in areas surrounding the hole in control samples, especially on the exterior side. In the IP sample a considerably larger volume of new mineralized tissue has formed in the same areas, as well as on the interior side. Figure 6 also shows for comparison a sample from an animal that died in surgery (Day 0).

### Quantification of bone formation and remodeling by micro-CT

**Figure 7:**
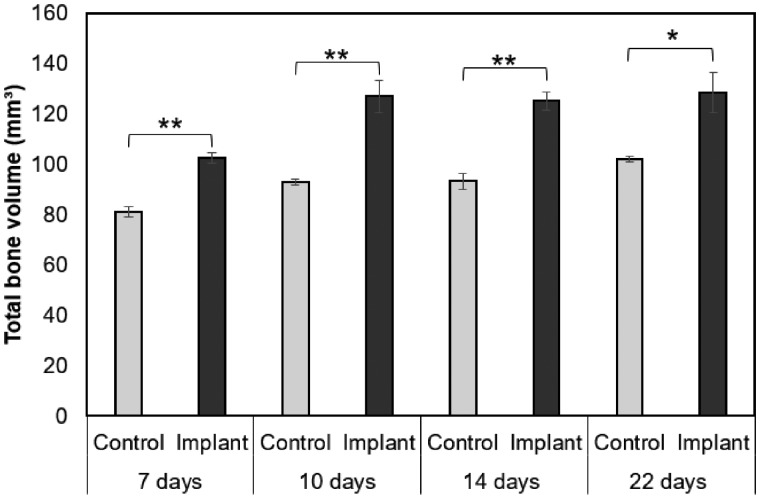
Change in total bone volume in a defined part of rat mandible as a function of time. Results from two-tailed *t*-test, statistical significance between control and implant groups for each time is indicated (*P < 0.05, **P < 0.01). Graphs represent mean±SEM

Since new bone was forming in the vicinity of the IP but not in the IP itself a different approach than originally planned was taken to analyze the samples, looking at a larger volume around the IP rather than the IP itself. New bone appears darker than old bone on micro-CT images, therefore, these can be separated using a defined grayscale value. However, for our study the IP had similar grayscale value distribution as new bone. Therefore, the IP volume was subtracted and TBV outside of the IP was calculated (see Fig. 2 and Materials and methods). Changes in TBV were rapid during the first 10 days in all rats but after that, a plateau was reached in both groups (Fig. 7). From Days 7 to 10 the control group showed a 15% increase in TBV and the experimental group showed a 24% increase. According to a two-tailed *t*-test, statistically significant increase in TBV between control and IP was observed at all time points, with *P* < 0.01 for 7, 10 and 14 days and *P* < 0.05 for 22 days.

### Degree of acetylation affects bone remodeling

**Figure 8:**
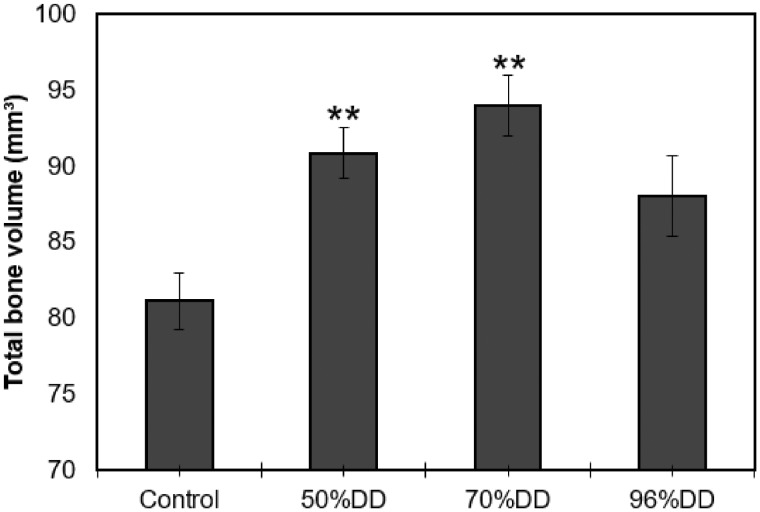
Change in total bone volume in a defined part of rat mandible as a function of implant type (%DDA). Results from one-way ANOVA comparing all treatment groups to the control (**P < 0.01). Graphs represent mean±SEM

To extend these findings, verify the model and examine how acetylation of the chitosan polymer affected the results, another experiment was performed. Experimental procedure was identical except here a single time point of 7 days was used and the IP volume was decreased from 40 to 25 µl. The test groups included a control group with an empty drill hole and three IP groups with a chitosan polymer of various degree of deacetylation, 96% DDA, 70% DDA and 50% DDA. TBV was calculated for each group (Fig. 8). The largest volume increase relative to control was in the 70% DDA group. The TBV was 16% higher than in the control group. The difference between the 50% DDA group and control was 12% but the 96% DDA group did not show a significant increase in the TBV in 7 days relative to control. According to a one-way analysis of variance (ANOVA), a significant difference in TBV was observed between control and IP with 50% DDA as well as between control and IP with 70% DDA, with *P* < 0.01. The difference in TBV between IPs with 50% DDA and 70% DDA was not statistically significant as judged by a *t*-test.

## Discussion

The findings of the present study are that a calcium phosphate-based IP containing 50–70% DDA chitosan polymer may stimulate new bone growth. However, new bone growth was not observed in the IP itself but rather in surrounding bone tissue. Three-dimensional representations produced from the X-ray micro-CT data showed new bone growth formed as an outgrowth from the periosteum, covering the surface of the bone, located along particular areas of the mandible. This finding suggests that it is the biomechanical weakening in the structure of the mandible that stimulates the osteogenesis observed in the present material. The rats cannot shield the mandible as they need to feed, therefore, there is a greater need to compensate for loss of mechanical strength or stability produced by the drill hole. In the time course experiment this compensation was observed in both empty drill hole controls and animals which received the 70% DDA chitosan and calcium phosphate IP. The important finding here is that this response was stronger and faster in the IP group. We further show that an IP with a 50% DDA chitosan polymer showed similar results as an IP containing 70% DDA chitosan polymer but an IP comprising 96% DDA chitosan polymer was not as effective. It can be speculated that the chitosan is increasing or stimulating a natural response to the structural weakening of the mandible and that the acetyl groups in the chitosan polymer play a role.

Chitinases cleave chitin and partly deacetylated chitin, but not fully deacetylated chitosan. Chitinases, including human chitinases (Chitotriosidase) need at least two consecutive acetylated glucosamine units (AA) on the partly deacetylated chitin polymer in order to cleave it into oligomers, resulting in AA at the reducing end of each oligomer product [15–17]. Chitinases and chitinase like proteins are highly expressed during inflammation [18], e.g. by immune cells such as PMN leukocytes. Therefore it is likely that the exposed, partly deacetylated chitosan polymer (50 and 70% DDA) is cleaved, forming oligomers locally during the inflammation phase in the drillhole wound, as the IP is slowly disintegrated. The highly deacetylated chitosan (96% DDA) is likely to be sparsely cleaved. The degree of deacetylation of chitooligosaccharides controls the binding affinity to the chitinase like protein YKL-40 and the effect on human chondrocyte proliferation [19]. Fully deacetylated hexamer binds very poorly to YKL-40 [19]. Chitosan having different degree of deacetylation has been shown to have different biological activity, where lower degree of deacetylation (higher acetylation; more chitin like) has the tendency to have stronger bioactivity. When comparing the effect of fully acetylated and deacetylated chitooligosaccharides (Hexamers) on osteogenic differentiation of bone-marrow derived human mesenchymal stem cells, the effect of chitooligosaccharides was dependent on the acetylation degree, with significantly stronger effects with chitin-derived hexamers (*N*-acetyl chitohexaose; fully acetylated) than with chitosan hexamers (chitohexaose; fully deacetylated) [20].

Our histological analysis shows that the 70% DDA chitosan IP attracts PMN leukocytes and we know that a 70% DDA chitosan polymer is a good chitinase substrate and will produce chitooligosaccharides of various sizes [21]. Therefore, it is possible that the IP is acting as a reservoir, allowing slow release of chitooligosaccharides which can diffuse from the IP and stimulate new bone growth in the periphery of the IP [21]. The reason the 96% DDA chitosan is not as effective might be because it is not a good substrate for chitinases, with only 4% of the units being *N*-acetyl-glucosamine.

When we compare our results to the literature it is surprising that we do not see any activity in the IP itself. However, the duration of this study might not be long enough to see osteogenesis in the IP. Furthermore, the IP is non-load bearing, and so does not impart the mechanical signal needed for active bone formation and remodeling. Other published studies generally allow more time to pass before checking for bone growth [12]. The peripheral bone growth has not been reported by other groups researching bone regenerative effects of chitosan IPs/scaffolds. One reason might be that this would generally get overlooked by researchers that are focusing on the IP itself. Another reason might be that the rat mandible model is especially sensitive to mechanical stress induced bone remodeling since the mandible cannot be shielded, even after an injury, because the animals need to feed. On the other hand, it might also be because at later time points this bone has been reabsorbed. At the time points that we selected, not enough time has subsided for the IP to be replaced by new bone, therefore, compensations need to be made in other areas of the mandible. As time passes bone might replace the IP and then the reinforcements around the bone defect might no longer be needed, perhaps causing resorption of the extra bone generated at earlier time points. The answer to these speculations can only be acquired by additional studies.

For clinical use in bone defects, the results point to the option of developing a chitosan containing scaffold or a composite IP that takes up mechanical load within the defect, and facilitates cell ingrowth, in order to stimulate osteogenesis within the defect.

## Conclusions

The results of the present study suggest that the bone regenerating effect of mechanical stimulation of the bone tissue is increased by chitosan or chitooligosaccharide containing IPs.

The effect on bone regeneration also depends on the degree of deacetylation of the chitosan used.

Use of chitosan-calcium phosphate soft composites as bone void fillers is not feasible for short-term stimulation of osteogenesis within a void. For restoration of function, a soft composite may still be feasible as a reservoir of chitosan, accelerating osteogenesis in the vicinity of the IP site.

## References

[1] Oryan A, Alidadi S, Moshiri A et al Bone regenerative medicine: classic options, novel strategies, and future directions. J Orthop Surg Res 2014;9:18. 24628910 10.1186/1749-799X-9-18 PMC3995444

[2] Dimitriou R, Jones E, McGonagle D et al Bone regeneration: current concepts and future directions. BMC Med 2011;9:66. 21627784 10.1186/1741-7015-9-66 PMC3123714

[3] Dennis SC, Berkland CJ, Bonewald LF et al Endochondral ossification for enhancing bone regeneration: converging native extracellular matrix biomaterials and developmental engineering in vivo. Tissue Eng Part B Rev 2015;21:247–66. 25336144 10.1089/ten.teb.2014.0419 PMC4442558

[4] Sheikh Z, Javaid MA, Hamdan N et al Bone regeneration using bone morphogenetic proteins and various biomaterial carriers. Materials (Basel) 2015;8:1778–816. 28788032 10.3390/ma8041778 PMC5507058

[5] Carragee EJ, Hurwitz EL, Weiner BK. A critical review of recombinant human bone morphogenetic protein-2 trials in spinal surgery: emerging safety concerns and lessons learned. Spine J 2011;11:471–91. 21729796 10.1016/j.spinee.2011.04.023

[6] Collignon AM, Lesieur J, Vacher C et al Strategies developed to induce, direct, and potentiate bone healing. Front Physiol 2017;8:927. 29184512 10.3389/fphys.2017.00927 PMC5694432

[7] Venkatesan J, Vinodhini PA, Sudha PN et al Chitin and chitosan composites for bone tissue regeneration. Adv Food Nutr Res 2014;73:59–81. 25300543 10.1016/B978-0-12-800268-1.00005-6

[8] Younes I, Rinaudo M. Chitin and chitosan preparation from marine sources. Structure, properties and applications. Mar Drugs 2015;13:1133–74. 25738328 10.3390/md13031133 PMC4377977

[9] Klokkevold PR, Vandemark L, Kenney EB et al Osteogenesis enhanced by chitosan (poly-N-acetyl glucosaminoglycan) in vitro. J Periodontol 1996;67:1170–5. 8959566 10.1902/jop.1996.67.11.1170

[10] Pang Y, Qin A, Lin X et al Biodegradable and biocompatible high elastic chitosan scaffold is cell-friendly both *in vitro* and *in vivo*. Oncotarget 2017;8:35583–91. 28103580 10.18632/oncotarget.14709 PMC5482600

[11] Ho MH, Yao CJ, Liao MH et al Chitosan nanofiber scaffold improves bone healing via stimulating trabecular bone production due to upregulation of the Runx2/osteocalcin/alkaline phosphatase signaling pathway. Int J Nanomedicine 2015;10:5941–54. 26451104 10.2147/IJN.S90669 PMC4590342

[12] Levengood SL, Zhang M. Chitosan-based scaffolds for bone tissue engineering. J Mater Chem B Mater Biol Med 2014;2:3161–84. 24999429 10.1039/C4TB00027G PMC4078888

[13] Oryan A, Sahvieh S. Effectiveness of chitosan scaffold in skin, bone and cartilage healing. Int J Biol Macromol 2017;104:1003–11. 28684351 10.1016/j.ijbiomac.2017.06.124

[14] Eliaz N, Metoki N. Calcium phosphate bioceramics: a review of their history, structure, properties, coating technologies and biomedical applications. Materials (Basel) 2017;10:334. 10.3390/ma10040334 PMC5506916 28772697

[15] Eide KB, Norberg AL, Heggset EB et al Human chitotriosidase-catalyzed hydrolysis of chitosan. Biochemistry 2012;51:487–95. 22192075 10.1021/bi2015585

[16] Bahrke S, Einarsson JM, Gislason J et al Sequence analysis of chitooligosaccharides by matrix-assisted laser desorption ionization postsource decay mass spectrometry. Biomacromolecules 2002;3:696–704. 12099813 10.1021/bm020010n

[17] Haebel S, Bahrke S, Peter MG. Quantitative sequencing of complex mixtures of heterochitooligosaccharides by vMALDI-linear ion trap mass spectrometry. Anal Chem 2007;79:5557–66. 17595055 10.1021/ac062254u

[18] Kzhyshkowska J, Gratchev A, Goerdt S. Human chitinases and chitinase-like proteins as indicators for inflammation and cancer. Biomarker Insights 2007;2:128–46. 19662198 PMC2717817

[19] Einarsson JM, Bahrke S, Sigurdsson BT et al Partially acetylated chitooligosaccharides bind to YKL-40 and stimulate growth of human osteoarthritic chondrocytes. Biochem Biophys Res Commun 2013;434:298–304. 23541584 10.1016/j.bbrc.2013.02.122

[20] Lieder R, Thormodsson F, Ng C-H et al Chitosan and chitin hexamers affect expansion and differentiation of mesenchymal stem cells differently. Int J Biol Macromol 2012;51:675–80. 22790025 10.1016/j.ijbiomac.2012.07.005

[21] Aam BB, Heggset EB, Norberg AL et al Production of chitooligosaccharides and their potential applications in medicine. Mar Drugs 2010;8:1482–517. 20559485 10.3390/md8051482 PMC2885077

